# Lack of neo-sensitization to Pen a 1 in patients treated with mite sublingual immunotherapy

**DOI:** 10.1186/1476-7961-8-4

**Published:** 2010-03-15

**Authors:** Renato E Rossi, Giorgio Monasterolo, Cristoforo Incorvaia, Philippe Moingeon, Franco Frati, Giovanni Passalacqua, Lucilla Rossi, Giorgio W Canonica

**Affiliations:** 1 Allergy Unit, National Health Service, Rete di Allergologia Regione Piemonte, Cuneo 1, Italy; 2 Dipartimento di Analisi Chimico-Cliniche e Microbiologia, Ospedali di Fossano e Savigliano, Italy; 3 Allergy/Pulmonary rehabilitation, ICP Hospital, Milan, Italy; 4 Research and Development, Stallergènes SA, Antony, France; 5 Medical and Scientific Department, Stallergenes, Milan, Italy; 6 Allergy and Respiratory Diseases DIMI, University of Genoa Italy; 7 Laboratorio Centrale di Analisi, Istituto Gaslini, Genoa, Italy

## Abstract

**Background:**

Some studies reported the possible induction of food allergy, caused by neo-sensitization to cross-reacting allergens, during immunotherapy with aeroallergens, while other studies ruled out such possibility.

**Objectives:**

The aim of this study was to evaluate the development of neo-sensitization to Pen a 1 (tropomyosin) as well as the appearance of reactions after ingestion of foods containing tropomyosin as a consequence of sublingual mite immunization.

**Materials and methods:**

Specific IgE to Tropomyosin (rPen a 1) before and after mite sublingual immunotherapy in 134 subjects were measured. IgE-specific antibodies for mite extract and recombinant allergen Pen a 1 were evaluated using the immunoenzymatic CAP system (Phadia Diagnostics, Milan, Italy).

**Results:**

All patients had rPen a 1 IgE negative results before and after mite SLIT and did not show positive shrimp extract skin reactivity and serological rPen a 1 IgE conversion after treatment. More important, no patient showed systemic reactions to crustacean ingestion.

**Conclusions:**

Patients did not show neo-sensitization to tropomyosin, a component of the extract (namely mite group 10) administered. An assessment of a patient's possible pre-existing sensitisation to tropomyosin by skin test and/or specific IgE prior to start mite extract immunotherapy is recommended.

**Trial Registration:**

This trial is registered in EudraCT, with the ID number of 2010-02035531.

## Introduction

In developed countries respiratory allergy is an important cause of chronic illness [1] and has a significant socio-economic impact [2]. As stated in consensus documents, allergen specific immunotherapy (SIT) is the only curative approach to treat respiratory allergic diseases [3,4]. As a matter of fact, commercial extracts used for SIT contain all or almost all the sensitizing molecules, including major and minor allergens, whereas patients receiving the treatment may be sensitized to only some of them. This raises the question of whether the administration of allergen extracts during immunotherapy can induce new clinically relevant sensitizations [5], that is the appearance of IgE specific for other allergenic molecules. Some studies have, in fact, reported the development of new sensitizations to allergenic components during subcutaneous immunotherapy (SCIT) with grass pollen [6] and birch pollen [7], but the clinical impact of such new sensitizations remained unexplored. On the other hand, there have been convincing reports about the onset of clinical manifestations of food allergy to snails or crustaceans in patients receiving SLIT with mite extracts [8,9]. This effect can be attributable to the emergence of specific IgE to tropomyosin, a group 10 component of the mite extracts, which is shared also by snails, shrimps and lobsters. In the case of shrimps, tropomyosin is the major allergen Pen a 1 [10-13]. Thus, it is important to assesss if de-novo sensitisation to tropomyosin occurs during mite immunotherapy, and if such sensitisation maybe of clinical relevance. The present study was designed to assess in real life the development of new sensitizations to Pen a 1 (tropomyosin), and the onset of reactions after ingestion of foods containing tropomyosin, as a possible consequence of sensitization to this allergen (Group 10, i.e. Der p 10) contained in the mite extracts for SIT [11,13].

## Materials and methods

### Patients and immunotherapy

Consecutive patients with ascertained respiratory allergy due to house dust mite, and prescribed with mite sublingual immunotherapy (SLIT) were evaluated at baseline (before starting the SLIT courses) and after three years of treatment, concerning their sensitisation to shrimp and positivity to Pen a 1. The prescription of mite-SLIT was made according to guidelines, in the presence of a case history consistent with mite allergy, that is symptoms of persistent rhinoconjuntivitis and/or mild to moderate asthma for at least 2 years. In addition they had to have a positive skin prick test to house dust mite extract and/or a positive CAP-RAST assay (>.35 kU/L). Exclusion criteria were previous treatment with SLIT or severe or uncontrolled asthma. SLIT was given as a standardized mite extract (Staloral^®^, Stallergenes, Antony, France) at the concentration of 60 μg/ml Der p 1, 12 μg/ml Der p 2 and 150 μg/ml Der f 1. The content of group 10 allergen (tropomyosin), was as high as 19 μg/ml. Matched patients receiving SLIT for grass (Staloral 300^®^) for a cumulative dose of 7,560 μg of Phl p 5 in three years, were the control group. In addition, patients with ascertained reactions to shrimp were studied for their skin and in vitro reactivity to shrimp and mite, as positive controls. The outpatients were enrolled during the period from January 2005 to December 2007. All subjects or their parents signed an informed consent.

### *In vivo* tests

Skin prick tests were performed with commercial extracts of dust mites (Alk-Abellò, Lainate, Milan, Italy) containing 40 μg/ml of Der p 1 and Der f 1 and 20 μg/ml of Der p 2 and Der f 2. No information was given by the manufacturer about mite group 10 (tropomyosin) content. In addition, the following allergen extracts from the same manufacturer were used: *Parietaria judaica *(20 μg/ml Par j 1), *Phleum pratense *(60 μg/ml Phl p 5), birch (45 μg/ml Bet v 1), olive (60 μg/ml of Ole e 1), mugwort (135 μg/ml Art v 1) and cat dander (60 μg/ml Fel d 1). All patients were also skin tested with a shrimp extract (Stallergenes) before and after SLIT.

### *In vitro* tests

IgE-specific antibodies for mite extract and recombinant allergen Pen a 1 obtained from *Penaeus aztecus *was evaluated using the immunoenzymatic CAP system (Phadia Diagnostics, Milan, Italy) according to the manufacturer's instructions. The results were expressed in classes of positive results from 0 to 6, where class 0 corresponds to up to 0.35 kUA/l; class 1 to 0.35-0.7 kUA/l; class 2 to 0.7-3.5 kUA/l; class 3 to 3.5-17.5 kUA/l; class 4 to 17.5-50 kUA/l; class 5 to 50-100 and class 6 more than 100 kUA/L.

## Results

One hundred and thirty-four patients were enrolled for the study, received SLIT for their mite allergy and completed the 3-year course of treatment. The mean age of the patients was 29.4 years, and 64 of them were male. The control group included 155 subjects (mean age 27.1 years, 83 male) who received SLIT for grass allergy. The two populations were homogeneous, for demographic and clinical characteristics, as shown in table 1. At baseline, serum IgE specific to Pen a 1 were undetectable in all the 288 subjects, and the skin prick test with the commercial shrimp extract was negative. After 3 years of SLIT with the mite extract, there was no emergence of Pen a 1-specific IgE as measured by CAP-RAST. Also, the skin test performed with the shrimp extracts remained negative in the two groups of subjects. There was no change in the pattern of skin sensitisation to mite and other allergens at the end of the SLIT course. Of the 134 patients receiving mite SLIT, all had certainly ate crustaceans (including shrimp) and seafood on more occasions during SLIT, and none of them reported adverse reactions. The same happened in the patients of the control group. In 6 patients with previous reactions after shrimp ingestion, the skin test with shrimp extract proved invariably positive, as well as the CAP assay to shrimp and the measurement of Pen a 1 specific IgE (Table 2). This showed that the shrimp extract efficiently detects the Pen a 1 positive subjects.

**Table 1:** Demographic, clinical and serological characteristics of 182 patients treated with sublingual vaccines

	Mite SLIT	Grass SLIT
Number of patients	134	155

Age (years), mean ± SD	29.4 ± 14.1	27.1 ± 13.8

Male (%)	64 (48)	83 (53)

Female	70 (52)	72 (47)

Rhinitis (%)	73 (54)	99 (63)

Rhinitis+Asthma (%)	61 (46)	56 (37)

Pen a 1-negative subjects	134	155

Mean SLIT duration (months)	24.7 (min 12 - max 36)	36 (min 36-max 36)

**Table 2:** Characteristics of the shrimp-allergic patients

Patient	1	2	3	4	5	6
Age	8	11	9	49	27	14

Skin test to mite (mm)	8	7	11	6	8	5

Skin test to shrimp (mm)	7	6	8	6	6	7

sIgE to mite (kUA/l)	23.76	>100	12.81	4.77	37.11	93.7

sIgE to shrimp (kUA/l)	15.64	>100	6.93	5.77	23.56	83.29

sIgE Pen a 1 (kUA/l)	18.15	>100	9.73	64.23	21.89	>100

## Discussion

The observations on development of new sensitizations induced by specific immunotherapy are contrasting. Studies demonstrating new IgE reactivities to allergenic components in pollen extracts administered with SLIT did not report association with clinical symptoms [6,7]. Concerning dust mite immunotherapy, the relevant allergen is tropomyosin, occurring in mites, crustacean and molluscs and in a number of other invertebrates [10-13]. A first study found that SCIT with dust mite extract induced an increase of IgE response to snail or shrimp and the occurrence of clinical symptoms following their ingestion [8]. However, only in two patients, who were negative at baseline to tropomyosin, a new sensitization actually took place. There are reports that subjects already clinically allergic to foods containing tropomyosin may worsen their food allergy symptoms following mite immunotherapy [14,15], but this is a different issue. Indeed, in a study on two groups of children, respectively treated or not by mite immunotherapy for a mean duration of 19 months, the only new sensitization to snail was found among children not treated with immunotherapy [16]. Of course, the eating habit is an important factor: in the study by Meglio et al less than 20% of children had previously eaten snails [16], but in the study by Asero et al all the included patients ate crustaceans and molluscs of any kind with no clinical reaction [9]. It is also true that a sensitisation to shrimp's tropomyosin can be found in subjects allergic to mites who never ate shrimps or shellfish [17].

We studied patients living in a geographic area in the province of Cuneo in northwest Italy, where molluscs are widely consumed as part of the local gastronomic tradition. There are two small cities specifically within the area, Borgo San Dalmazzo and Cherasco, where the consumption of snails in particular is quite high; snails are even the subject of frequent local culinary festivals. All patients had a personal histories free from systemic allergic reactions to crustaceans and molluscs (oysters, snails, prawns, lobster etc.). The main finding of our study is that all 134 patients with negative rPen a1 IgE before starting immunotherapy, remained tropomyosin IgE negative after completing the mite immunotherapy course. Of course, an assessment of Der p 10-specific IgE would have been more specific, but such reagent was not available at the time of the study. In addition, the shrimp skin test and shrimp-specific IgE assay proved positive in shrimp-allergic patients, so that the reliability of those tests in detecting positive subjects is reasonably ascertained.

To our knowledge the present paper is the first one in which the evaluation of a large number of patients treated with mite sublingual/oral vaccines did not result in a neo-sensitisation to tropomyosin, which was significantly present (as mite group 10) in the extract administered. This suggests that a persistent and continuative ingestion of a tropomyosin source as mite extract might not be enough to neo-sensitize mite allergic patients. The length of time a given subject receives SLIT seems also be a notable factor. The patients we studied had their treatment for a mean duration of more than 2 years, that may be considered a reasonable period in which a neo-sensitisation can occur. Although the occurrence of IgE reactivity to tropomyosin was reported in initial observations on patients treated with mite SCIT [8], a subsequent study specifically addressing the issue was unable to observe such phenomenon within a 3-year duration of mite SCIT [9]. Our findings demonstrate that also with prolonged SLIT neo-sensitisation to tropomyosin does not occur, and this was confirmed by negative skin tests to shrimp extract performed in all treated patients.

Obviously, the possibility of a neo-sensitisation to tropomyosin during SCIT or SLIT cannot be completely excluded. However, it is possible that occurrence of side effects induced by crustaceans or molluscs ingestion during mite immunotherapy could be due to lack of information about a pre-existent state of sensitisation to tropomyosin in these patients. To avoid possible allergic reactions to tropomyosin containing foods, we believe that assessment of a pre-existing sensitisation to tropomyosin by skin test and specific IgE measurement should be performed prior to start mite extract immunotherapy.

## Consent

Written informed consent was obtained from the patient for publication of this article. 

## Competing interests

The authors declare that they have no competing interests.

## Authors' contributions

RER conceived of the study, participated in its design, carried out the tests, analysed the results and participated to writing the manuscript

GM participated in the study design, carried out the tests, and analysed the results

CI analysed the results and participated to writing the manuscript

PM analysed the results and participated to writing the manuscript

FF participated in the study design, analysed the results and participated to writing the manuscript

GP analysed the results and participated to writing the manuscript

LR carried out the tests and analysed the results

GWC analysed the results and participated to writing the manuscript

All authors read and approved the final manuscript.
